# Length of residence, age and patterns of medicinal plant knowledge and use among women in the urban Amazon

**DOI:** 10.1186/1746-4269-10-25

**Published:** 2014-02-24

**Authors:** Coral Wayland, Lisa Slattery Walker

**Affiliations:** 1 Department of Anthropology, University of North Carolina Charlotte, 9201 University City Blvd, Charlotte, NC 28223, USA; 2 Department of Sociology, University of North Carolina Charlotte, 9201 University City Blvd, Charlotte, NC 28223, USA

**Keywords:** Medicinal plants, Age, Urban, Migration, Brazil, Amazon

## Abstract

**Background:**

This paper explores patterns of women’s medicinal plant knowledge and use in an urban area of the Brazilian Amazon. Specifically, this paper examines the relationship between a woman’s age and her use and knowledge of medicinal plants. It also examines whether length of residence in three different areas of the Amazon is correlated with a woman’s use and knowledge of medicinal plants. Two of the areas where respondents *may* have resided, the jungle/*seringal* and farms/*colonias*, are classified as rural. The third area (which all of the respondents resided in) was urban.

**Methods:**

This paper utilizes survey data collected in Rio Branco, Brazil. Researchers administered the survey to 153 households in the community of Bairro da Luz (a pseudonym). The survey collected data on phytotherapeutic knowledge, general phytotherapeutic practice, recent phytotherapeutic practice and demographic information on age and length of residence in the seringal, on a colonia, and in a city. Bivariate correlation coefficients were calculated to assess the inter-relationships among the key variables. Three dependent variables, two measuring general phytotherapeutic practice and one measuring phytotherapeutic knowledge were regressed on the demographic factors.

**Results:**

The results demonstrate a relationship between a woman’s age and medicinal plant use, but *not* between age and plant knowledge. Additionally, length of residence in an urban area and on a colonia/farm are not related to medicinal plant knowledge or use. However, length of residence in the seringal/jungle is positively correlated with both medicinal plant knowledge and use.

**Conclusions:**

The results reveal a vibrant tradition of medicinal plant use in Bairro da Luz. They also indicate that when it comes to place of residence and phytotherapy the meaningful distinction is not rural versus urban, it is *seringal* versus other locations. Finally, the results suggest that phytotherapeutic knowledge and use should be measured separately since one may not be an accurate proxy for the other.

## Background

It was early Wednesday morning when fourteen-month-old Thales woke the entire household with his insistent crying. When his mother, Graça, pulled him out of the hammock she noticed that he was running a slight fever. Then, as she rocked him back to sleep, Thales began to vomit. To lower his fever Graça gave him a tea brewed from orange leaves. Later that day, Thales developed diarrhea. During that day and the next, Graça and Thales’s grandmother, Adriana, continued to treat him with home remedies made from medicinal plants. They used various teas to alleviate his condition: orange leaf tea to lower his fever, mint tea to soothe his upset stomach and a tea brewed from guava leaves to stop his diarrhea.

*When Thales showed no signs of improvement after two days of treatment with home remedies, Graça and Adriana took him to a prayer healer. Both Adriana and Graça agreed that Thales’s symptoms disappeared after the prayer ceremony and they believed he was cured. However, when Thales awoke on Sunday he was once again suffering from a high fever, chills, vomiting and diarrhea. Graça took him to the Hospital de Base, or public emergency care facility, where he was intravenously rehydrated and given pharmaceuticals*^*a*^*. His condition improved and he was sent home. Thales then took another turn for the worse on Monday. He returned to the Hospital de Base where he was once again intravenously rehydrated and given pharmaceuticals. After another seven days of home treatment with pharmaceuticals from the hospital and home remedies made from medicinal plants, Thales finally recovered*.

This passage from the first author’s fieldnotes illustrates the various treatment options (home remedies, pharmaceuticals, and consultations with prayer healers and clinicians) that the urban poor in Amazonia use when they fall ill. It also highlights the importance of one option, *remédios caseiros*, or home remedies made from medicinal plants. Medicinal plants are often the first line of defense against common health problems among Rio Branco’s low income population [1]. While Graça and Adriana utilized a number of different treatment options, they initially attempted to manage Thales’s illness using home remedies. Moreover, caregivers generally use phytotherapy throughout an illness episode even when they employ other treatment options. In this example, Thales’ caregivers continued to use medicinal plants throughout his illness even after they consulted a prayer healer and biomedical professionals.

As the example above suggests, medicinal plant use is one of many treatment modalities used in the *urban* Amazon. Most discussions about medicinal plant knowledge and use in the Amazon, however, refer to indigenous or forest dwelling groups, such as *caboclos* and rubber tappers [2-17]. There has been little research documenting medicinal plant use among urban Amazonian populations (for exceptions see [1,18-20]). This paper seeks to redress this lack of knowledge by examining patterns of medicinal plant use among a non-indigenous population in a peri-urban slum in the city of Rio Branco, Brazil.

One of the reasons ethnobotanists have begun to study medicinal plant use in urban areas is because urban migration and the acculturation that accompanies it may lead to changes in patterns of medicinal plant knowledge and use (e.g. [21,22]). In many *rural* Amazonian areas, physical isolation and financial poverty mean phytotherapy may be one of the few options to treat health problems [23,24]. When populations leave rural areas for the city, however, they live closer to pharmacies, doctors, clinics and hospitals. This may lead migrants to utilize more biomedical remedies (and perhaps fewer phytotherapeutic ones) [17,25,26]. Upon arriving in a city migrants are also exposed to modern lifestyles, which many increasingly embrace. Consequently, some urban migrants may gradually abandon plant remedies in favor of biomedical health care (e.g. [25,27,28]). Additionally, in urban areas younger generations who have little to no experience in rural areas may reject plants in favor of pharmaceuticals. Finally, it is important to understand patterns of phytotherapy in urban areas because the practice can lead to herb-drug interactions when used in conjunction with pharmaceuticals, which are often easier to come by in cities.

This paper contributes to the growing body of literature on medicinal plant use in urban areas by exploring patterns of phytotherapeutic knowledge and practice in the Amazonian city of Rio Branco. Specifically, it examines the relationship between medicinal plant knowledge/use, women’s age, and length of residence in urban and rural areas. This research sought to examine two scenarios about medicinal plant use in urban areas. The first is that younger women would have less knowledge about medicinal plants and be less likely to use medicinal plants. The second is that length of residence in an urban area is negatively associated with medicinal plant use and knowledge. The results of this study demonstrate a relationship between a woman’s age and medicinal plant use, but *not* between age and plant knowledge. The results did not reveal a significant relationship between urban residence and phytotherapeutic use or knowledge. They did, however, reveal that length of residence in the jungle (*the seringal*) was positively correlated with both medicinal plant knowledge and use in our sample.

### Research setting

Before discussing the results, it is necessary to situate this analysis in a historical, political, and economic context. Prior to the 1960s, 79% of the residents in the Amazonian state of Acre lived in the forest (*seringal*) [29]. These rural residents could roughly be divided into two groups: indigenous populations (which are not discussed in this paper) and rubber tappers, or *seringueiros* as they are also known. Due to the virtual absence of primary health care in the rural Amazon, *seringueiro* households are self-sufficient in terms of illness management. While *seringueiro* households do have recourse to specialized healers like midwives and prayer healers, their main method of coping with illness is self-treatment with medicinal plants, at times supplemented with Western pharmaceuticals. Indeed, biomedical health care is often sought only in cases of extreme illness [13,23,25]. Due to their heavy reliance on phytotherapy, most *seringueiro* caregivers are knowledgeable about medicinal plants [10,30].

This rural lifestyle began to change in the 1970s when the Brazilian government embarked on a policy of directed development in the Amazon. This involved the clearing of large tracts of forest to develop farming communities, known as *colonias*, which led to the increase of a second type of rural resident, small scale farmers known as *colonos*^b^. In Acre, most of the families who moved into *colonias* were former *seringueiros*, while a minority were peasant farmers from southern Brazil. Like rubber tappers, *colonos* also relied heavily on medicinal plants to manage illness episodes.

By the late 1970s ranchers and speculators were moving into Acre and expelling small scale farmers and rubber tappers from rural areas [31-34]. During this time landless settlers from other Amazonian states also began arriving in Acre. With nowhere to go, the dispossessed and newly arrived migrants moved to cities, fueling rapid and unplanned urbanization in the Amazon [35-38]. By 1990 over 60% of the population in the Brazilian Amazon resided in an area classified as urban [35].

In Rio Branco (the state capital of Acre), most migrants moved into one of the numerous peri-urban communities that checker the city’s landscape [39,40]. These communities formed on the outskirts of the city or along flood plains when uninhabited tracts of land are “claimed” by low income households during coordinated land grabs. These low income households usually engage in a number of revenue generating and subsistence strategies, many of which are part of the informal economy. Most households make between 1-2 minimum wages, depending on the number of wage earners. As this process continues and urban areas continue to grow these neighborhoods shift from being on the periphery of an urban area to being more centrally located.

The neighborhood of Bairro da Luz (a pseudonym), where research was conducted, was typical of these peri-urban communities. By 1996 Bairro da Luz encompassed approximately 350 households. Most houses were one-room wooden dwellings occupied by entire families. In 1996, Bairro da Luz was still lacking municipal garbage collection, piped water or city sewer connections. Pirated electricity was the one basic service available to community residents.

When Brazilian citizens experience health problems, they are entitled to free health care at any public health facility. Unfortunately, Rio Branco’s public health system is under-funded, under-equipped, and under-staffed [19]. These factors limit the availability and quality of curative care available in Rio Branco’s public health care facilities. Consequently, while public health care is free, it is often unavailable or substandard.

Medicinal plant use is one way caregivers in peri-urban households cope with this lack of public health care. Households gain access to medicinal plants by growing them on their small urban lots. Almost all of the houses in the neighborhood have a yard (mostly dirt). Many residents grow a variety of plants in these spaces, including medicinals and a few sources of food. Most medicinal plants are grown in dooryard gardens (in the ground), though on occasion some residents plant them in containers. At the time research was conducted the roads and paths in Bairro da Luz were not paved and were hemmed in on both sides by weeds. While some of these weeds may have had phytotherapeutic properties residents did not report collecting plants from open spaces in the neighborhood. Instead, families who did not cultivate their own medicinal plants had access to them in their neighbors’ gardens. Indeed, phytotherapeutic material and knowledge are shared among friends, neighbors, and relatives in many parts of the Amazon [1,41,42].

## Methods

### Data collection

The data in this paper were collected during a survey conducted in the community of Bairro da Luz, (a pseudonym), located in Rio Branco, Brazil. The first author and two Brazilian research assistants administered the survey. Participant informed consent was obtained from all participants. A sample of 153 households (44% of the houses in the neighborhood) completed the survey. Starting with the first house on each street, every other occupied house was included in the survey. When the interviewers encountered a house that was empty or whose residents were temporarily residing elsewhere, the adjacent house was selected. When the interviewers encountered an occupied house where nobody was home, they returned three more times: once during the day, once at night, and once on a weekend. If after the fourth attempt nobody was contacted, the house was not included in the survey. Previous ethnographic fieldwork in the area indicated that women were primarily responsible for managing illness episodes among household members [1]. As a result, interviewers spoke with the “*dona da casa*” or woman of the house in each of the 153 households. As such, this represents a meaningful sample that can be generalized to the neighborhood. Generalization to larger populations, however, must be made carefully.

The survey contained 22 structured questions that collected data on phytotherapeutic knowledge, general phytotherapeutic practice, recent phytotherapeutic practice and demographic information. Data on phytotherapeutic knowledge were gathered by having women free-list plants used to treat the following conditions: fever, respiratory infections, diarrhea, gynecological problems, contraception, to induce menstruation (women report that they induce menstruation to maintain health, not just to terminate unwanted pregnancies), and wound healing [27,43]^c^. These seven conditions were selected because previous ethnographic research indicated they were the most common health problems reported by these households [40]. Having women free-list plants for specific illnesses standardized their responses because they responded to the same prompts (instead of thinking of illnesses themselves, which would vary from woman to woman). Women were also directed to list the plants they knew for each condition, regardless of whether they used the plant or grew it. Finally, at times women would name one plant for more than one illness. For example, orange leaf was frequently listed as a cure for fever and for respiratory infections. As such, 18 total responses (or *5, 6, 7*, etc.) does not necessarily represent 18 different plants.

Data on general phytotherapeutic practice were elicited by asking how frequently women used medicinal plants to treat household members, how often they purchased medicinal plants, where they learned to use medicinal plants, whether they ever grew medicinal plants, whether they thought plants or pharmaceuticals were stronger, whether they were currently growing plants, and, if so, how many plants were currently being grown. The demographic data for each respondent include the woman’s age and length of residence in a city, on a farm (*colonia*), and in the jungle (*seringal*). All data are self-reported.

Data on recent phytotherapeutic practice were collected by asking women whether anyone residing in their house had been ill in the seven days prior to the interview. A seven day recall period was selected to minimize the problems of recall bias that lead women to over or underestimate general measures of phytotherapeutic practice. Consequently, all women were asked if any member of the household had been ill in the past seven days. If a household member had been ill, the woman was asked whether medicinal plants had been used to treat the individual.

### Data analysis

Descriptive statistics were calculated for measures of phytotherapeutic knowledge, general phytotherapeutic practice, and recent therapeutic practice. Multivariate analyses attempting to explain variance in medicinal plant knowledge and use were then created. Bivariate correlation coefficients were calculated to assess the inter-relationships among the key variables. Then, three dependent variables, two measuring general phytotherapeutic practice (frequency of use of medicinal plants measured on a likert-type scale and the number of medicinal plants currently being grown in the household garden) and one measuring phytotherapeutic knowledge (the total number of medicinal plants named for the set of specific conditions) were regressed on the demographic factors. These factors include the length of time the respondent had lived in each of three areas (urban, *colonia* and *seringal*). Age is not included in the regression analyses due to its high correlation with the number of years lived in the three areas.

Research was approved by the University of Pittsburgh Institutional Review Board.

## Results

### Descriptive statistics

#### Phytotherapeutic knowledge

**Figure 1:**
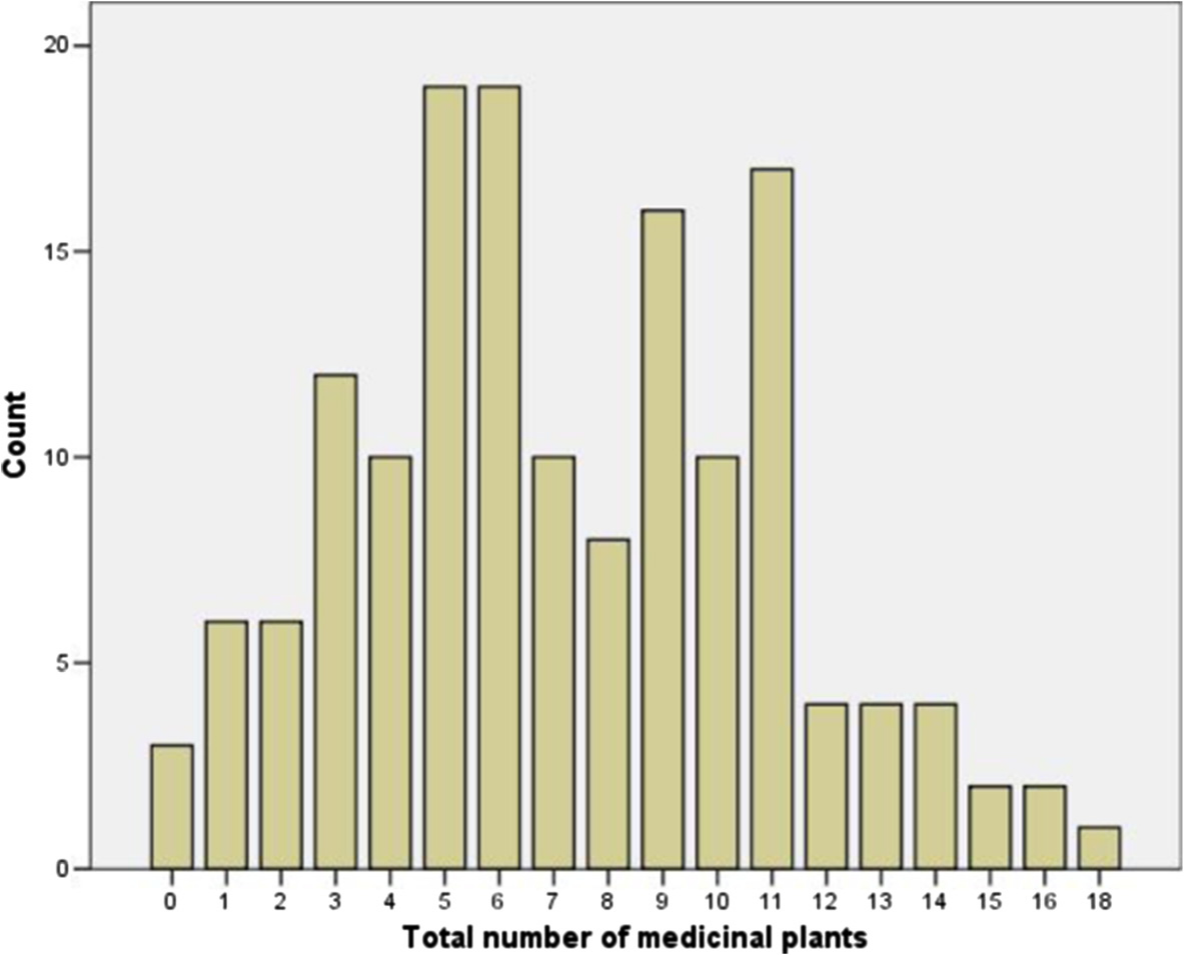
Number (count) of women who named each number of medicinal plants named overall (for all conditions).

Medicinal plant knowledge was measured by asking women to free-list plants for fever, respiratory infections, diarrhea, gynecological problems, contraception, to induce menstruation, and wound healing. As such, the results below do not represent the total number of medicinal plants women are familiar with. There was wide variation in the number of plants women listed, ranging from 0 to 18 (Figure 1). Only three women could not name any plants, six women could name only one plant, and six women could name only two plants.

**Figure 2:**
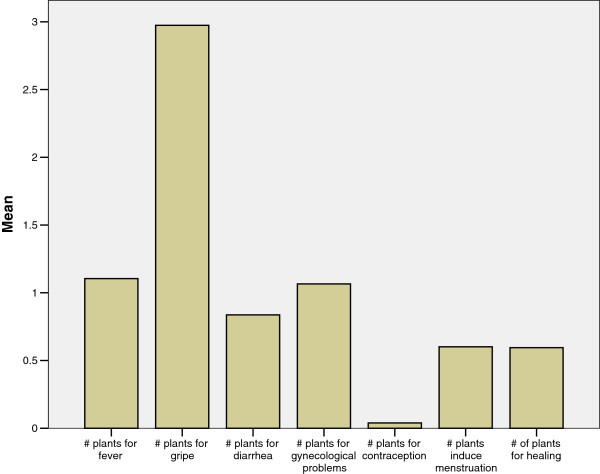
Mean number of medicinal plants named for specific conditions.

Women could name the most plants (an average of three) for the treatment of respiratory infections (*gripe*). This is not surprising given how common respiratory infections are, especially among children. One study found that 36% of children experienced one or more of the following symptoms in the 7 days preceding the survey; wet or dry cough, nasal congestion, or difficulty breathing [40]. On the other hand, women could name very few plants used for contraception (well less than one on average). For each of the other five health conditions (fever, diarrhea, gynecological problems, to induce menstruation and wound healing) women could name an average of one plant each (Figure 2).

The survey also asked women “Which do you think is stronger, medicinal plants, pharmaceuticals, or it depends on the illness?” While this is not a measure of phytotherapeutic knowledge, it does provide insight into how women think about medicinal plants. Nearly 60% of women believe that medicinal plants are stronger than pharmaceuticals^d^. Only about 25% believed that pharmaceuticals were stronger, with the remaining 15% said it depends on the condition.

#### General phytotherapeutic practice

The survey also contained questions about phytotherapeutic practice in general. One measure of general phytotherapeutic practice is how often women reported using medicinal plants to treat their own and their family members’ health problems. When asked this question, 41% of the women responded "always," 11% "almost always," 28% "occasionally," 16% "rarely," and only 4% reported "never" used phytotherapy. This yields a total of 80% of respondents who use medicinal plants at least occasionally.

It is also interesting to note where women report learning about medicinal plants. Seventy nine percent of women report that they learned about phytotherapy from family members and 11% learned from friends and neighbors. Only 10% resorted to more formal methods like books, courses or lectures. While this measure does not control for age, many of the 90% of women whose family, friends and neighbors taught them about medicinal plants are undoubtedly “younger” women. In fact, the average age of women who reported learning from family and friends is not different from those who learned from more formal sources (t = -.691, n.s.).

**Figure 3:**
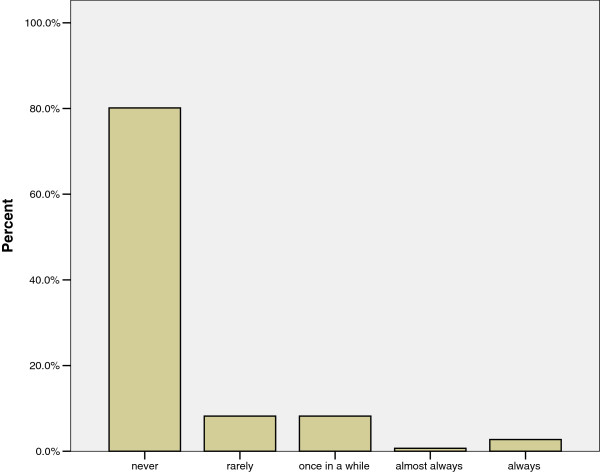
Frequency that women report purchasing medicinal plants from a market downtown.

A final element of general phytotherapeutic practice is where women obtain medicinal plants. While medicinal plants are sold at the downtown market, few women (only about 20%) avail themselves of this option (Figure 3). The women who do purchase plants report doing so rarely (40%) or infrequently (40%). Instead, most women get the plants that they use from their gardens or the gardens of friends, neighbors and relatives. Over 70% of women claim to cultivate medicinal plants in their household gardens and nearly 60% had plants growing in their gardens at the time of the survey. The discrepancy was due mainly to two factors. First, it was the dry season so some plants had died and women were waiting for the rains to start before they replanted. Second, some women had recently moved to the area and had not yet planted anything or they were waiting for the rainy season.

#### Recent phytotherapeutic practice

To get data about actual phytotherapeutic practice, the survey collected information about medicinal plant use during the seven days prior to the interview. Eighty households reported having at least one member who had been ill during the past 7 days. Of these 80 households, 50% had used medicinal plants to treat the illness. Of the 50% of households that did not use phytotherapy, 20% said that they either planned to use phytotherapy in the future (i.e. the illness was very recent) or they had planned on using phytotherapy but the illness resolved spontaneously before they made the remedy. Only 30% did not use phytotherapy nor did they plan on using it prior to the resolution of the illness episode.

### Bivariate and multivariate analyses

Descriptive statistics for the key variables in our bivariate and multivariate analyses are presented in Table 1. The three key dependent measures are: how often medicinal plants are used, the number of medicinal plants that are currently cultivated in the garden, and the total number of medicinal plants listed to treat the seven specific conditions named in the survey. The first two are measures of practice and the third is a measure of knowledge.

**Table 1:** Descriptive statistics for key variables

	**N**	**Minimum**	**Maximum**	**Mean**	**Standard deviation**
How often do you use medicinal plants? (1, never to 5, always)	152	1	5	3.70	1.265
Number of medicinal plants in the garden	153	0	8	2.31	2.718
Total number of plants named	153	0	18	7.22	3.777
Age	153	14	84	31.50	12.534
Place of birth (1 = Acre)	152	0	1	0.81	0.394
Number of years in urban areas	147	1	47	16.79	8.922
Number of years in a colonia	147	0	39	4.57	6.708
Number of years in seringal	148	0	44	9.79	11.156

Frequency of medicinal plant use is coded from 1, “never,” to 5, “always.” The number of medicinal plants in the garden and the total number of plants named are counts. The mean for how often plants are used is 3.70, indicating that women on average use plants more frequently than “once in a while,” but less than “almost always.” The average number of medicinal plants growing in their gardens is 2.31 and the average number of plants they could name to treat the whole list of conditions was 7.22.

The demographic independent variables include the woman’s age, the length of time in any urban area, length of time on a farm (*colonia*), and length of time lived in the forest (*seringal*). The women ranged from 14 to 84 years old, with an average age of 31.50 years. Some of the women had never lived on a farm or in the *seringal*, and some had been in an urban area for as little as a few months. The average number of years lived on a farm and in the *seringal* were 4.57 and 9.79, respectively. The average number of years spent living in an urban area was 16.79. Simple bivariate correlations (Table 2, Pearson’s zero-order correlation coefficients reported) show that the three dependent variables are significantly positively correlated with one another. Not surprisingly this indicates that women who have knowledge of medicinal plants also tend to grow and use them.

**Table 2:** Bivariate correlation coefficients (N)

	**How often do you use medicinal plants?**	**Number of medicinal plants in the garden**	**Total number of plants named**	**Age**	**Number of years in urban areas**	**Number of years in a colonia**
How often do you use medicinal plants?						
Number of medicinal plants in the garden	.265**					
	(152)					
Total number of plants named	.328**	.496**				
	(152)	(153)				
Age	.205*	.214**	.156			
	(152)	(153)	(153)			
Number of years in urban areas	-.014	-.069	.007	.192*		
	(146)	(147)	(147)	(147)		
Number of years in a colonia	.047	.104	.081	.360**	-.277**	
	(146)	(147)	(147)	(147)	(147)	
Number of years in seringal	.221**	.247**	.127	.663**	-.403**	.007
	(147)	(148)	(148)	(148)	(147)	(147)

*p < .05.

**p < .01.

#### Age

The correlations between age, plant use and plant knowledge yield mixed results. The two dependent practice measures, growing plants in the garden and using medicinal plants, are significantly correlated with age. The older a women is the more likely she is to use and grow medicinal plants. However, medicinal plant *knowledge* is not significantly correlated with age. Younger women are no less (or more) able to name medicinal plants used to treat specific conditions than are older women.

This study also compared the ages of women that think medicinal plants are stronger than pharmaceuticals, and vice versa. The average age of women who think medicinal plants are stronger is 31.22, while the average of those who think pharmaceuticals are stronger is 31.43 (t = -.088, n.s.). Thus, younger women are no more likely than older women to say that pharmaceuticals are stronger than medicinal plants.

#### Residence

The bivariate correlations of the dependent variables with residence reveal that neither the amount of time spent living in a *colonia* (farm) nor the amount of time living in an urban area are correlated with any of the dependent measures. People who have spent decades living in a *colonia* are not more (or less) knowledgeable about phytotherapy, do not use more (or less) medicinal plants, and do not grow more (or less) plants than people who have never lived on a farm. The same is true for the amount of time spent living in a city. The amount of time spent living in the *seringal*, however, is positively correlated with both use of medicinal plants and growing of plants. Time in the *seringal* is not correlated with medicinal plant knowledge (as was the case with age). This means that while women who have spent more time living in the *seringal* are more likely to practice phytotherapy and grow plants than those who have spent less time in the forest, they do not list more medicinal plants.

Because length of residence in each area is correlated (i.e., the more time you have lived in one area the less time you have lived in the others), multivariate analysis is used to test the independent effects of each residence variable holding constant the effects of other variables. This way we can disentangle the effect of each of the three places of residence on medicinal plant knowledge and use. Regression analyses for each of the three dependent measures are presented in Table 3. The independent variables are the same for each model: number of years lived in an urban area, number of years lived on a farm, and number of years lived in the *seringal*. Age is not included as an independent variable since it is equivalent to the sum of the three independent variables regarding length of residence and would cause issues of multi-collinearity. Ordinary least-squares (OLS) regression is used for the number of plants growing and the number of plants named, as these are continuous count variables. OLS is appropriate as there is no reason to assume the dependent variables have non-normal distributions in the population from which this sample is drawn. Multinomial logistic regression is used for frequency of plant use since it is a categorical independent variable.

**Table 3:** Regression analyses

	**How often do you use medicinal plants?**	**Number of medicinal plants in the garden now**	**Total number of plants named**
	**-2 Log-likelihood**^**a**^	**B (standard error)**	**B (standard error)**
Intercept	377.652	1.320 (0.825)	6.133 (1.151)
Place of birth (1=Acre)	373.631	-0.312 (0.564)	-0.432 (0.787)
Number of years in urban areas	373.753	0.024 (0.028)	0.044 (0.040)
Number of years in a colonia	374.726	0.049 (0.035)	0.059 (0.048)
Number of years in seringal	380.940*	0.068 (0.022)**	0.056 (0.030)^
	Psuedo-R^2^ (Cox and Snell) =.143, N=146	R^2^=. 077, N=146	R^2^=.032, N=146

^p < .10.

*p < .05.

**p < .01.

^a^Chi-square statistic were calculated to determine significant effects. The chi-square statistic is the difference in -2 log-likelihoods between the final model and a reduced model. The reduced model is formed by omitting an effect from the final model. The null hypothesis is that all parameters of that effect are zero.

The number of years lived in the *seringal* is the only independent variable to retain a meaningful connection to the dependent variables. Women who have lived in the *seringal* are more likely to use medicinal plants. The more time a woman has spent in the *seringal*, the more likely she is to use medicinal plants and grow them in her garden, our measures of general phytotherapeutic practice. She may also be able to name more plants for specific conditions, indicating greater phytotherapeutic knowledge as well. For each additional 10 years spent in the seringal, women grow and can name, on average, one additional plant.

The total explained variance for these models is relatively low, indicating that, as a group, our independent variables do not explain much of the variability in responses to our dependent measures. Only between 3 percent and 14 percent of the variation in plant knowledge and use is explained by our included variables. Thus, there may be other, as yet unmeasured, variables which are driving these relationships, such as family experiences and level of medical need. It may be that urbanism, in particular, is not as connected to phytotherapeutic knowledge and practice as previously believed. Time in the city or on a *colonia* seems to be less important than time in the jungle.

## Discussion

These results contribute to a growing body of literature that expands our understanding of patterns of medicinal plant knowledge/use in urban areas [22,25,27,44]. Contrary to some statements (e.g. those made by physicians in Rio Branco), phytotherapy is still widely practiced in Bairro da Luz [45]. Women are knowledgeable about medicinal plants and many (60%-75% depending on the illness) believe that plants are better than or on equal footing with pharmaceuticals. Women also continue to use plants and many cultivate plants in their gardens. Finally, according to the women interviewed, the tradition of using plants continues to be passed on. This suggests a dynamic tradition of medicinal plant use in this urban environment.

One of the questions this study was interested in exploring was whether younger urban residents were less likely to know about and use medicinal plants than older residents. The answer to this question is nuanced. The positive correlation between medicinal plant use and age has been noted elsewhere [46]. However, the finding that younger women seem to know as much as older women is at odds with studies in other areas of the world [25,27,47-49]. There have been, however, some studies that note a similar lack of a relationship between age and medicinal plant knowledge [50-52]. One possible explanation that reconciles these two observations has been suggested elsewhere [4,25,27,46]. Many older women have larger families and/or more health problems. These facts may lead them to utilize medicinal plants more frequently. Since they use plants more often they also grow more of them. Indeed the results that link age to ideas about efficacy lends support to this interpretation (lower level of use is not due to a lack of knowledge or belief in efficacy, but likely due to lower need).

The second question this paper was interested in exploring was whether urban residence was negatively correlated with phytotherapuetic practice and knowledge. The results indicate that it is not. In this analysis length of urban residence has no demonstrable effect on medicinal plant use or knowledge. This suggests that as people spend more time in a city, which may lead them to adopt more a more urban outlook and lifestyle, they do not necessarily abandon (either suddenly or gradually) phytotherapy. In this case a focus on *urban* migration (and the changes that come with it) as factors contributing to the disappearance of phytotherapy may be slightly off the mark [49,53]. It is not moving to a city that leads to decreased medicinal plant knowledge and use. It is leaving the *seringal*. This suggests the need to move beyond a simple urban vs. rural distinction when examining the relationship among place of residence and phytotherapy.

In this analysis the meaningful distinction is not rural versus urban, it is *seringal* versus other locations. While length of residence in each area is certainly related (the longer you live in one, the less time you live in another), the fact that length of residence in the *seringal* is the only significant variable, is an important distinction to make. Some might argue that urbanization is the flip side of the depopulation of the *seringal* (when people leave the *seringal* they go to a city), but this is not always the case in Acre. People can and do move from the *seringal* to *colonias*. However, length of residence in a *colonia* has no demonstrable effect on plant knowledge or use. It seems that in this context a focus on “de-seringalization” is more appropriate than a focus on urbanization. This indicates that future studies should take care to distinguish between different types of rural residence.

This research also revealed that practice and knowledge variables are correlated with age and residence in a very interesting way. Social scientists have long known that people do not always act on the knowledge they possess. In Bairro da Luz this seems to be the case with phytotherapy. While use and cultivation of medicinal plants increase with a woman’s age and with length of residence in a seringal, knowledge of medicinal plants does not. These results are in line with other studies that indicate the need to disentangle these two aspects of phytotherapy in future studies on the status of medicinal plants [54-56]. Phytotherapeutic knowledge and use should be measured separately since one may not be an accurate proxy for the other.

## Endnotes

^a^We use the terms pharmaceuticals and drugs to refer to medications dispensed in biomedical health care facilities and purchased in pharmacies. They contrast with phytotherapeutic options such as home remedies (*remédios caseiros*).

^b^By definition the seringal and colonias are always rural. In some areas, however, colonias are located in close proximity to peri-urban areas of cities. At the time research was conducted the closest colonias were located about 20-30 minutes (by vehicle) outside of Rio Branco.

^c^Specific species are not discussed in this paper because voucher specimens were not expected.

^d^The survey used the word “forte”, which translates to English as strong. Previous ethnographic research with this population found that this is used as a gloss that can mean different things, including overall efficacy or the speed at which plants/pharmaceuticals work.

## Competing interests

The authors declare that they have no competing interests.

## Authors’ contributions

C W oversaw data collection and data entry. She also bore the ultimate responsibility for drafting and revising the manuscript. LS W ran the statistical analysis and wrote the statistical portions of the manuscript. Both authors read and approved the final manuscript.
